# Assessment of variations in productive performance of two different plumage color varieties of Japanese quail and their reciprocal crosses

**DOI:** 10.1007/s11250-023-03604-5

**Published:** 2023-05-05

**Authors:** Ibrahim Elkhaiat, Seham El-Kassas, Yahya Eid, Magda Ghobish, Esteftah EL-Komy, Mahmoud Alagawany, Mohamed Ragab

**Affiliations:** 1 Department of Poultry Production, Faculty of Agriculture, Kafrelsheikh University, Kafr El Sheikh, 33516 Egypt; 2 Animal, Poultry and Fish Breeding and Production, Department of Animal Wealth Development, Faculty of Veterinary Medicine, Kafrelsheikh University, Kafr El Sheikh, 33516 Egypt; 3 Animal Production Department, Agriculture and Biology Research Division, National Research Centre, El Buhouth St., 12311 Dokki, Cairo, Egypt; 4 Department of Poultry, Faculty of Agriculture, Zagazig University, Zagazig, 44511 Egypt

**Keywords:** Japanese quail, Plumage color mutations, Genetic diversity, Microsatellite markers, Body weight, Carcass traits, Egg quality

## Abstract

This study aimed to detect the phenotypic differences between the brown (BB) and white (WW) feathered quails and their reciprocal crosses (BW and WB) over two successive generations. The WW and cross quails, especially the BW, had the heaviest body weights, throughout the studied period, with significant variations between the two studied generations (*P*<0.05). Moreover, the WW and BW possessed the largest egg production during the F1, while in the F2, the BB had superiority among the studied quails with a prominent superiority of the F2 over the F1 (*P*<0.05). However, the F1 had higher egg weights than F2 with superiority of WW quails compared to the others (*P*<0.05). Also, the WW quails had the lowest lipid contents of the eggs. These phenotypic variations among the studied quails might be preliminarily explained by the results of the analyzed microsatellite markers despite the few markers used. The high variability among the BW and WB quails might be due to the larger number of alleles (*N*_*A*_ and *N*_*e*_) and the lower values of *F*_IS_ with low heterozygosity levels (*H*_*O*_ and *H*_*e*_). Moreover, the BW and BB were the closest, while WB and WW were the farthest because of the high and low genetic identities and the high and low genetic distance between them, respectively. So the obtained results might introduce an initial scientific basis for evaluating and employing the genetic properties of BB, WW, BW, and WB quails in further genetic improvement program, and more microsatellite markers are recommended.

## Introduction

Japanese quail (*Coturnix japonica*) is a small promising poultry species reared mainly for meat and egg production, especially in rural low-resource areas. It is characterized by distinct productive and reproductive characteristics including rapid growth, short generation interval, early sexual maturation, stress and disease resistance, and a high rate of laying eggs (Farajiarough et al. 2018).

As a result of plumage color mutations, there are many well-known varieties of Japanese quails such as gray-, white-, and brown-colored-feathered quails (Bagh et al. 2016; ELSaidy et al. 2021). These varieties have distinguished productive and reproductive characteristics (Ibrahim et al. 2021). Accordingly, quail varieties are divided into meat-producing, egg-producing, or dual-purpose quails (Ibrahim et al. 2021). Because of the close breeding within the same population, the Japanese quail is very susceptible to inbreeding depression, so crossbreeding between the distant quail varieties is effective to restore the genetic diversity in quail populations (Nunome et al. 2020). Consequently, it is crucial to assess the physiological and genetic variations among these varieties to establish efficient breeding strategies to improve their production by providing the needed information about the most effective genotypes (Dávila et al. 2009).

Microsatellite markers or simple sequence repeats (SSRs) are good genetic markers that can be effectively used to study the genetic variance within or among breeds (Nunome et al. 2020). They are characterized by the presence of 1–6 base repeats in the eukaryotic genome including birds (Kayang et al. 2000), abundant polymorphism, codominance inheritance, and easy scoring by the polymerase chain reaction (PCR) (Kayang et al. 2002). In quail, microsatellites can be used to establish quail genetic maps. In this regard, Kayang et al. 2004 (Kayang et al. 2004) established the first generation of microsatellite markers linkage map which contains 72 microsatellite markers. Additionally, Miwa et al. (2005) fixed the position of quail functional genes and QTLs depending on three microsatellite primers: GUJ0071, GUJ0097, and GUJ0061 (Miwa et al. 2005). Moreover, microsatellites can be effectively used to analyze the genetic diversity among different quail populations through polymorphism analysis (Bai et al. 2018).

Despite that there are many studies that have reported the variation in the productive and reproductive traits among different quail varieties, they only documented this at the phenotypic level, and only the genetic parameters have been calculated. Besides, to the best of our knowledge, only one study (Ibrahim et al. 2021) has involved the genetic diversity analysis depending on microsatellite markers, and this was done on different quail varieties. Thus, the current study aimed to illustrate the physiological variations in two varieties of Japanese quails (brown-feathered quail strain (BB), white-feathered quail strain (WW), and their reciprocal crosses (BW×BW and WB×WB). Besides, we tried to provide a preliminary explanation and understanding of the genetic bases of these variations using some microsatellite markers.

## Material and methods

### Bird management

The current study was conducted on four populations of Japanese quail (*Coturnix japonica*): brown-feathered (BB) and white-feathered quail (WW) varieties and their reciprocal crosses (BW×BW and WB×WB). The bird management was done following the regulations of the animal care and ethics committee of the Poultry Production Department, Faculty of Agriculture, Kafrelsheikh University, Egypt. The study continued for two generations after establishing the base population. The number of chicks for each population and the number of selected groups during the laying period in the two generations are listed in Table 1.

**Table 1:** Total number of chicks in each line and the number of selected groups during laying period in three generations

	BXW		BXB		WXW		WXB	
Total number through the experiment								
F1	240		224		110		359	
F21	113		182		77		198	
F22	114		131		70		128	
Number of selected groups during laying period								
	F	M	F	M	F	M	F	M
F0	112	56	84	42	64	32	132	66
	BW×BW		BB×BB		WW×WW		WB×WB	
	F	M	F	M	F	M	F	M
F1	60	30	78	39	50	25	72	36
F21	34	17	56	28	32	16	42	21
F22	40	20	34	17	30	15	36	18

One-day-old chicks were wing-banded and weighed before being moved to heated brooders where they were reared on the floor in a conventional open-sided house until they reached 6 weeks old. Thereafter, they were individually housed in cages (depth: 15 cm; width: 18 cm; height: 18 cm).

The house was equipped with electric and gas heaters where the temperature was kept at 35 °C through the first 3 days and 32 °C during the next 4 days and step by step decreased by 2 °C per week until it was kept at 24 °C. Birds received a 24-h lighting photoperiod for the first 2 weeks depending on the natural and artificial light, followed by a 16 h:8 h light-to-dark cycle. Food and water were offered ad libitum. Birds were fed a standard diet of 28% crude protein (CP), 3100 kcal metabolizable energy (ME) kg^−1^ from 0 to 2 weeks, followed by a grower diet (24% CP and 2900 kcal ME kg^−1^) from 4 to 6 weeks of age. During the laying period, birds received a layer diet of 20% CP and 2700 kcal ME.

### Phenotypic traits

Body weight **(**BW) was recorded weekly from the beginning to 6 weeks old and then at 12 and 15 weeks old. The eggs (EN) laid from the 6th week until 15 weeks old were individually recorded. Egg weight (EW) was also individually recorded to the nearest 0.1 g for 3 days/week for each hen, and the average egg weight was determined per hen.

To assess egg quality, egg weight, albumen height, albumen width, albumen weight, yolk width, yolk height, yolk color, yolk weight, the Haugh unit (HU), and eggshell thickness were recorded individually, where yolk and albumen height were recorded using a tripod micrometer (mm). To evaluate the egg shape index, two axes, transversal and longitudinal of the egg, were measured using the Vernier caliper, and then, the egg shape index was calculated depending on the following formula: $\textrm{Egg}\ \textrm{shape}\ \textrm{index}=\frac{\textrm{transversal}\ \textrm{axis}}{\textrm{longitudinal}\ \textrm{axis}}\times 100$. Albumen and yolk indices were calculated from their height and width measurements using the following formula:

$$\textrm{Albumen}\ \textrm{or}\ \textrm{yolk}\ \textrm{index}=\frac{\textrm{Height}}{\textrm{Width}}\times 100.$$

At 47 days old, through the F1 and F2, 20 males and 20 females from each quail population were randomly selected and euthanized by cervical dislocation after 12 h fasting according to ELSaidy et al. (ELSaidy et al. 2021) to determine carcass traits including weight after slaughtering, carcass weight (empty carcass), and relative organ weights (%), including the liver, gizzard, heart, intestine, abdominal fat, ovary, testicle, and oviduct.

### Biochemical analysis

At the end of the second generation (F2), blood samples (*n*=20/group) were collected from the wing vein into tubes without anticoagulant, allowed to coagulate at room temperature, and then centrifuged at 3000×*g* for 10 min to separate the serum, which was stored at −20 °C for further analysis. Total cholesterol, creatinine, total antioxidant, lipid peroxide (malondialdehyde), albumin, and total lipid concentrations were colorimetrically determined using an automatic analyzer (ChemWell®, Megazyme, Wicklow, Ireland) and commercial kits (Labtest Diagnóstica S. A, Lagoa Santa, Brazil).

### Microsatellite markers isolation and genotyping

The analysis was carried out at the Animal Production Biotechnology Laboratory, Central Lab Network, National Research Center, Dokki, Egypt. A volume of 3 mL of whole blood was collected from the wing veins of 20 individuals from each quail population (BB = B×B, WW = W×W, BW = BW×BW, and WB = WB×WB). Blood samples were collected in sterile tubes containing 0.5 mL EDTA as an anticoagulant and stored at −20 °C until DNA extraction. Genomic DNA was extracted using GeneJET Whole Blood Genomic DNA Purification Mini Kit (Thermo Fisher Scientific) according to the manufacturer’s instructions. The integrity of DNA was electrophoretically evaluated using 1% agarose gel stained with ethidium bromide (0.5 μg/mL). DNA concentration and purity were assessed using the NanoDrop (Eppendorf, Hamburg, Germany) with absorbance at a wavelength of 260 nm and 280 nm.

Three microsatellite markers were used to recognize the variations of phenotypic traits between the four populations of Japanese quail. Full characterization of the analyzed microsatellite markers was presented in a previous study by Kayang et al. (Kayang et al. 2000). One monomorphic (GUJ0096) and two polymorphic markers (GUJ0063 and GUJ0085) were considered in this study. The primer sequences of these three microsatellite markers with their annealing temperature, GenBank accession numbers, and their length of repeats are shown in Table 2. The PCR mixture was carried out in a total volume of 25 μL containing 50 ng genomic DNA, 10 pmol of each primer, 2.5 μL 10X buffer, 1.5 mM MgCl_2_, 0.2 mM of each dNTP, and 1 U from DreamTaq (Thermo Fisher Scientific). The reaction was accomplished in TM Thermal Cycler (MJ Research PTC-100 thermocycler, USA). The thermal cycling profile included an initial denaturation step at 95 °C for 5 min followed by 35 cycles of 94 °C for 30 s, annealing at 55 °C for 1 min, and extension at 72 °C for 1 min, followed by a final extension step of final extension at 72 °C for 5 min. PCR successful products were identified on 2% agarose gel in 1X TBE buffer and visualized after staining with ethidium bromide (EtBr).

**Table 2:** Microsatellite loci, annealing temperatures, primers sequence, gene bank accession numbers, and repeated array and size range

Locus name	GenBank accession number	Repeat array	Forward primer (5_–3_)	Reverse primer (5_–3_)	Size range (bp)	TA (°C)
GUJ0063	AB063131	(CA)7CT(CA)2CT(CA)7	GCTCAGGTTCTCAGCTGATG	GGGAGAGATCAAGGGAACAG	242–250	55
GUJ0085	AB063153	(GT)14	ACAACCACTTCTCCAGCTAC	GCTTGTGCTGCTGTTGCTAA	245–265	55
GUJ0096	AB063164	(A)10(CA)14(A)20	GTACCAAAAGTGAATAGTGG	CAGATCACAGACTTAGAAAG	157	55

### Statistical analysis

GLM procedure of SAS 9.2 (SAS Institute Inc., 2008) was used to analyze the phenotypic data. All the traits during the fattening and laying periods were analyzed depending on the following linear model: *Y*_ijk_= *μ*+ *G*_*i*_ +GT_*j*_ + (*G*×GT)_ij_+ *e*_ijk_, where *μ* is the general mean, *G*_*i*_ is the effect of generation (2 levels (F_1_, F_2_), GT_*j*_ is the effect of genetic type or quail varieties, (*G*×GT)_ij_ is the effect of interaction between generation and quail varieties, and *e*_ijk_ is the residual effect. The different levels of each effect included in the models were compared using Duncan’s multiple range test where significance levels were detected as a first-class error at *α*=0.05. For carcass traits and blood parameters, the previous model was applied but with adding the sex effect (2 levels). For microsatellite data, the number of alleles for each marker was counted using a microsatellite analyzer 4.05 (Dieringer and Schlötterer 2003). Additionally, the mean number of effective alleles (*N*_*e*_, the number of equally frequent alleles at a marker) and the heterozygosity of the 3 SSRs (observed heterozygosity (*H*_*O*_), expected heterozygosity (*H*_*e*_)) in the four quail lines were estimated using GenAlEx version 6.0 (Peakall and Smouse 2006). Polymorphic information content (*P*_IC_) was determined using the Cervus software package (CERVUS 3.0.7). The fixation index (*F*_IS_) for each marker and the chi-square statistic for the Hardy-Weinberg equilibrium (HWE) were also calculated using the GENEPOP program version 3.4 (Raymond 1995). Also, using the POPULATIONS software (version 1.2.30) (Saitou and Nei 1987), the microsatellite phylogenetic tree of the four studied quail populations was constructed depending on the Nei genetic distance (DA) by using the neighbor-joining (NJ) method.

## Results

### Effect of quail varieties and generation of body weight measurements

Table 3 displays the effect of quail varieties, generation, and their interaction on body weight during the fattening and laying period. These factors significantly influenced the body weight measurements (*P* < 0.05). At the first week of age, the WB quail variety in F1 showed the highest body weights (*P* < 0.05). This effect was reversed in F2, and WB quails along with BW had the lowest body weights among the quail populations (*P* < 0.05). With increasing birds’ age (at the third and fourth weeks of age), the WW quails displayed the highest weights, followed by BW, compared to the other quail genotypes (*P* < 0.05).

**Table 3:** Body weight measurements among the four Japanese quail varieties (BB, WW, BW, and WB) during fattening period (W1, W3, and W4) and laying period (W6, W12, and W15) in two successive generations (F1 and F2). Data was presented as μ

	Groups	W1	W3	W4	W6		W12		W15	
					Males	Females	Males	Females	Males	Females
F1	BB	43.84±0.4^bc^	109.29± 1.1^de^	167.06± 1.5^e^	240.39±2.9^ab^	275.91±2.71^a^	259.85± 3.8^bcd^	300.08± 4.98^a^	267.06±5.2^abc^	323.03±5.42^a^
	WW	42.66±0.6^bc^	124.10±1.6^d^	188.66± 2.2^cd^	245 ±4.1^a^	291.00±4.32^a^	274.16± 5.3^a^	320.12±4.9^a^	262.15±13.6^abc^	332.96±5.37^a^
	BW	40.97±0.5^bc^	119.95± 1.2^de^	176.75± 1.9^de^	245.17±4.04^a^	284.65±2.64^a^	264.82±3.5^abc^	314.02±5.24^a^	260.17± 5.3^bc^	338.00±6.68^a^
	WB	53.66±0.5^a^	102.71±0.9^e^	175.20± 1.2^de^	223.61 ±2.1^d^	265.76±27.48^a^	269.68±3.9^ab^	307.02±4.00^a^	272.14± 4.8^ab^	336.50±4.68^a^
Overall		46.48±0.33	111.80±0.64	175.15±0.85	237.71±1.77	278.03±7.76	266.54±2.05	309.11± 2.43	265.59±3.48	332.13±2.84
F2	BB	40.42±0.4^bc^	154.14±1.3^c^	202.95± 1.5^bc^	235.27 ±2.98^abc^	257.44± 2.93^a^	258.38±3.2^cd^	304.68±3.19^a^	257.33± 3.54^bc^	314.70± 3.66^a^
	WW	46.06±0.7^b^	172.91±2.1^ab^	223.36± 1.9^a^	228.22±2.98^cd^	262.41±3.81^a^	252.02± 2.5^d^	308.93± 4.28^a^	251.71±2.88^c^	326.77±4.90^a^
	BW	38.91±0.5^bc^	155.32±1.4^c^	206.38± 1.6^bc^	241.5± 4.4^ab^	269.71±3.34^a^	273.44±3.96^a^	315.58±4.43^a^	278.12±5.03^a^	329.46±4.44^a^
	WB	37.76±0.3^c^	156.99±1.2^bc^	203.19± 1.4^bc^	232.95±2.4^bcd^	264.55±3.02^a^	259.07±3.02^bcd^	313.12±4.02^a^	271.28± 2.9^ab^	331.64± 4.25^a^
Overall		40.09±0.25	158.19±0.74	207.26±0.83	233.80±1.59	263.17±1.63	259.75±1.65	310.34±1.97	263.42±1.94	263.42±1.94
*P* values										
Genotype		<0.0001	<0.0001	<0.0001	0.0763	0.04	0.0122	0.93	0.8381	0.05
Generation		<0.0001	<0.0001	<0.0001	<0.0001	0.5339	<0.0001	0.0119	0.0170	0.0027
Genotype×generation interaction		<0.0001	<0.0001	<0.0001	0.0022	0.6451	0.6056	0.2543	0.0457	0.9769

Results were presented as mean ± SE. Different letters in columns indicate significant differences among the groups at *P* < 0.05

During egg production, the male’s and female’s weights were recorded (Table 3). At 6 weeks old, the WW and BW males, in the first generation, had significantly higher body weights compared to the other quail varieties (*P* < 0.05), while WB males had the lowest body weight (*P* < 0.05). In the second generation, BW and BB males displayed the highest weights, while WW males had the lowest weights (*P* < 0.05). At 12 weeks old, the highest weights were found in the case of WW, WB, and BW (in a descending manner), but the BB males showed the lowest weights (*P* < 0.05). In the second generation, the BW males were the heaviest among the other quails’ males (*P* < 0.05). The latter finding continued until the end of egg production with the BW being the heaviest followed by WB males (*P* < 0.05). Unlike males, females did not show any changes in their body weights through the reported egg production period (*P* > 0.05).

### The external and internal egg quality characteristics

Differences in egg numbers and weights among different quail populations through two successive generations were also reported (Tables 4 and 5). It seems that the number of eggs produced was significantly influenced by quail varieties and the generation. In this regard, the F1 WW and BW recorded a distinct higher onset of egg production compared to the other quails’ populations (*P* < 0.05). This higher egg number continued to the seventh week of egg production and then declined. In the second generation (F2), the BB achieved the highest number of eggs produced compared to the other quail varieties (*P* < 0.05). However, the production fluctuated between increases and decreases throughout the period of egg production. In general, there was a clear superiority of the F2 production compared to the F1 with the BB quail population being the most superior among quail populations. Besides, egg weights were influenced by the quail genotypes and generation (Table 5). The F1 showed higher egg weights compared to the F2 with the superiority being reported in the case of WW quails in F1 compared to others (*P* < 0.05).

**Table 4:** Average numbers of egg produced in the four Japanese quail varieties (BB, WW, BW, and WB) during laying period (from W1 to W8) in two successive generations (F1 and F2)

		W1	W2	W3	W4	W5	W6	W7	W8
F1	BB	1.20±0.2^c^	3.57±0.3^c^	3.97±0.3^b^	4.01±0.3^ab^	5.35±0.4^ab^	5.23±0.3^ab^	5.05±0.3^abc^	4.57±0.3^ab^
	WW	2.40±0.3^ab^	4.08± 0.4^bc^	4.38±0.3^ab^	4.44±0.4^ab^	5.48±0.3^ab^	5.34±0.4^ab^	5.20±0.3^ab^	3.74± 0.5^c^
	BW	2.20±0.3^b^	4.55±0.4^ab^	4.41±0.3^ab^	5.22± 0.3^a^	5.46±0.3^ab^	5.83±0.4^a^	5.40±0.4^a^	4.14±0.4^ab^
	WB	0.40±0.1^d^	3.34± 0.4^c^	4.58±0.3^ab^	4.59±0.2^a^	5.94±0.3^a^	5.51±0.5^ab^	5.05±0.4^abc^	4.65± 0.4^ab^
Overall		1.43	3.82	4.50	4.35	5.57	5.46	5.15	4.34
F2	BB	3.04±0.2^a^	5.27±0.2^a^	4.72±0.2^ab^	4.67±0.2^a^	5.70±0.1^ab^	5.81±0.2^a^	4.35±0.2^bcd^	4.78±0.3^a^
	WW	2.73±0.3^ab^	4.78±0.3^ab^	4.38±0.3^ab^	3.71±0.3^b^	5.30±0.2^abc^	5.11± 0.3^ab^	4.32±0.3^bcd^	4.87±0.3^a^
	BW	2.41±0.3^ab^	4.45±0.2^ab^	4.16±0.2^b^	3.72±0.2^b^	4.48±0.3^c^	4.62±0.3^b^	3.70±0.3^d^	5.18±0.3^a^
	WB	2.40±0.3^ab^	4.50±0.2^ab^	5.16±0.2^a^	4.69± 0.2^a^	4.85±0.2^bc^	5.38±0.2^ab^	4.11± 0.2^cd^	5.17±0.2^a^
Overall		2.66	4.77	4.63	4.25	5.10	5.27	4.12	4.99
*P* values									
Quail genotype		<0.0001	<0.0001	0.7228	0.3836	0.0189	0.2985	<0.0001	0.0023
Generation		<0.0001	0.1557	0.1339	0.0981	0.2246	0.7350	0.8985	0.3624
Genotype×generation interaction		<0.0001	0.0171	0.0035	0.0222	0.0222	0.0544	0.3634	0.4449

Results were presented as mean ± SE. Different letters in columns indicate significant differences among the groups at *P* < 0.05

**Table 5:** Weights of egg produced in the four Japanese quail varieties (BB, WW, BW, and WB) during laying period (from W1 to W8) in two successive generations (F1 and F2)

	Groups	W1	W2	W3	W4	W5	W6	W7	W8
F1	BB	10.78 ± 0.4^ab^	13.41 ± 0.3^a^	13.16 ± 0.3^ab^	13.81 ± 0.2^ab^	14.19± 0.3^a^	14.45 ± 0.3^a^	15.90±0.7^a^	14.22± 1.4^abc^
	WW	12.20± 0.4^a^	13.35± 0.2^a^	13.35±0.2^a^	13.93±0.2^a^	14.34± 0.2^a^	15.00 ± 0.3^a^	14.57± 0.2^abcd^	15.14±0.4^a^
	BW	11.46 ± 0.4^ab^	13.27 ± 0.2^a^	13.41 ± 0.3^a^	13.98 ± 0.5^a^	14.16± 0.2^a^	14.71 ± 0.3^a^	15.72± 1.4^ab^	14.49± 1.5^a^
	WB	10.41± 0.4^b^	13.39± 0.2^a^	13.41±0.3^a^	13.60± 0.3^abc^	14.41± 0.2^a^	14.62 ± 0.2^a^	15.24± 0.7^abc^	14.36±0.3^ab^
Overall		11.39	13.36	13.33	13.81	14.27	14.66	15.41	14.50
F2	BB	11.12± 0.2^ab^	12.08± 0.2^b^	13.08±0.2^a^	12.53±0.2^cd^	13.01±0.2^b^	13.15± 0.2^bc^	13.07±0.2^cde^	13.12±0.2^de^
	WW	10.73± 0.2^ab^	12.15± 0.3^b^	12.63±0.2^abc^	12.51±0.4^bcd^	12.76 ±0.1^bc^	12.68± 0.2^bcd^	12.37±0.3^e^	12.50±0.2^def^
	BW	11.41± 0.4^ab^	11.96± 0.2^b^	12.54±0.2^abc^	12.46±0.2^abcd^	12.64± 0.3^b^	12.99± 0.2^b^	12.90± 0.3^cde^	13.09±0.3^d^
	WB	10.45± 0.3^b^	11.55±0.2^b^	12.18±0.2^bc^	12.11±0.2^d^	12.45± 0.2^bc^	12.65± 0.2^bcd^	11.97± 0.2^e^	12.22±0.2^ef^
Overall		10.95	11.92	12.61	12.40	12.72	12.88	12.59	12.74
*P* values									
Quail genotype		0.3661	<0.0001	<0.0001	<0.0001	<0.0001	<0.0001	<0.0001	<0.0001
Generation		0.1154	0.5596	0.5881	0.5067	0.8056	0.7495	0.2339	0.1266
Genotype×generation interaction		0.0609	0.5186	0.0976	0.9726	0.3972	0.1461	0.8620	0.0164

Results were presented as mean ± SE. Different letters in columns indicate significant differences among the groups at *P* < 0.05

Examining the effect of quail varieties and generation differences on the external and internal egg characteristics revealed an obvious competition among quail varieties through the two generations (Table 6). The values of egg length and width in F2 were higher than those of F1 with the highest values measured in the case of BW for egg length and WB and BW for egg width (*P* < 0.05). Additionally, yolk and albumen heights demonstrated higher values in F2 compared to F1 with the BB and BW in F1 and WW and WB in F2 having the highest values (*P* < 0.05). For yolk width, the highest scores were reported in the case of BB in F1 compared to the other quail genotypes in the two generations (*P* < 0.05). Albumen length and width did not display any changes among different quail genotypes in the two generations (*P* > 0.05). For shell thickness, the F2 values were significantly higher compared to those of F1 with BB genotypes having superiority in the two generations (*P* < 0.05). The yolk color additionally exhibited marked changes (*P* < 0.05). The highest values were found in the case of WB in F1 and BB in F2. For egg shape and yolk indices, they did not show any significant changes among quail populations and between generations (*P* > 0.05). While the quail population caused a significant effect on the albumen index values, the greatest values were noticed in the case of the WB population in F2, and the lowest values were measured in the case of WW and WB in F1 (*P* < 0.05).

**Table 6:** Egg quality of the four Japanese quail vareities (BB, WW, BW, and WB) after four weeks of egg production

		Egg weight (g)	Egg length (cm)	Egg width (cm)	Yolk height (mm)	Albumen height (mm)	Yolk width (mm)	Albumen length (mm)	Albumen width (mm)	Shell thickness (mm)	Yolk color	Shape index	Albumin index	Yolk index
F1	BB	13.81 ±0.3^ab^	3.4 3 0.03^b^	2.66 ±0.02^abc^	12.54±0.3^ab^	5.08±0.4^b^	2.62± 0.04^a^	8.21±0.4^a^	6.62±0.26^a^	196.50±8.5^ab^	5±0.4^bcd^	77.81^a^	79.47^ab^	478.23^a^
	WW	12.68 ±0.29^c^	3.37 ± 0.02^b^	2.53± 0.05^d^	11.65±0.4^abc^	4.83±0.5^b^	2.48 ±0.03^ab^	9.29±0.29^a^	7.07±0.4^a^	177.00 ± 4.7^b^	4.9±0.3^cd^	75.04^a^	71.13^b^	467.87^a^
	BW	12.93±0.3^bc^	3.35 ± 0.04^b^	2.64 ± 0.02^abc^	12.09± 0.3^abc^	6.34± 0.4^a^	2.58±0.05^ab^	8.73± 0.58^a^	7.51±0.4^a^	177±5.58^b^	5.8±0.4^abcd^	79.05^a^	87.82^ab^	470.36^a^
	WB	12.46 ±0.27^c^	3.34 ± 0.03^b^	2.58± 0.02^cd^	11.07±0.2^c^	4.95±0.3^b^	2.54±0.04^ab^	9.39± 0.37^a^	6.99±0.4^a^	175.00 ±7^b^	6±0.4^ab^	77.31^a^	73.85^b^	435.80^a^
Overall		12.97	3.37	2.61	11.84	5.30	2.56	8.90	7.05	181.37	5.45	77.63	78.06	463.06
F2	BB	14.50 ±0.2^a^	3.46± 0.03^ab^	2.70± 0.02^ab^	11.78 ± 0.4^abc^	5.76±0.35^ab^	2.42±0.04^b^	8.87±0.4^a^	6.28±0.29^a^	234.72 ±15.2^a^	6.11±0.2^a^	78.13^a^	91.79^ab^	463.68^a^
	WW	14.57 ±0.2^a^	3.43 ± 0.05^b^	2.61 ± 0.04^bcd^	12.44±0.3^abc^	6.42±0.25^a^	2.45 ± 0.06^ab^	9.31±0.4^a^	6.87±0.39^a^	222.00± 12.4^a^	5.9±0.2^abc^	76.27^a^	101.19^ab^	513.46^a^
	BW	14.64±0.45^a^	3.59 ± 0.06^a^	2.74 ± 0.03^a^	11.14± 0.5^bc^	4.95±0.3^b^	2.49±0.04^ab^	9.16± 0.37^a^	7.15±0.56^a^	225.50 ±10.8^a^	4.75±0.3^d^	76.56^a^	76.62^ab^	450.10^a^
	WB	14.77 ±0.16^a^	3.48 ± 0.028^ab^	2.74± 0.01^a^	12.77±0.4^a^	6.61±0.36^a^	2.55± 0.06^ab^	8.57±0.4^a^	6.85±0.4^a^	221.76 ± 9.2^a^	5.7±0.3^abcd^	78.79^a^	104.59^a^	501.47^a^
Overall		14.62	3.49	2.69	12.01	5.91	2.48	9.00	6.80	225.93	5.60	77.33	93.15	481.90
*P* values														
Quail genotype		<0.0001	0.0009	0.0002	0.5397	0.0140	0.0520	0.8220	0.4436	<0.0001	0.4254	0.8747	0.0225	0.2563
Generation		0.3159	0.4492	0.0040	0.6475	0.8022	0.5126	0.4523	0.3342	0.4766	0.3348	0.2299	0.9076	0.6133
Genotype×generation interaction		0.0892	0.1461	0.3387	0.0099	<0.0001	0.3075	0.4204	0.9946	0.9778	0.0034	0.2777	0.0921	0.1844

Results were presented as mean ± SE. Different letters in columns indicate significant differences among the groups at *P* < 0.05

### Carcass trait properties among different quail populations throughout two successive generations

Differences in carcass characteristics among the four Japanese quail varieties and the two generations are presented in (Table 7). There are significant variations in live body weights and weights after slaughtering with the heaviest weights measured in the case of WB in F2 compared with the other varieties (*P* < 0.05). However, the highest percentages of carcass weights were noticed in the case of BW in F2, and WB showed the lowest % (*P* < 0.05). The relative weights of internal organs such as the liver, heart, and gizzard also exhibited marked differences among quail varieties and generations (*P* < 0.05). The highest relative weights of the liver were reported for WB in F1 and BW in F2 (*P* < 0.05), while the lowest relative weight was found in the case of WB in F2. The relative heart weight displayed the highest % in the case of BB in F2, while the lowest values were found in the case of BB, WW, and WB in F1 and WB in F2 (*P* < 0.05). For gizzard weights, there was not an obvious variation between F1 and F2, and the lowest % was found in the case of WB quail variety in F2 and WW in F1 (*P* < 0.05). The relative weight of the intestine was also calculated. All varieties exhibited nearly similar relative weights except WW which showed the lowest value (*P* < 0.05). BB and WB varieties in F2 showed the highest percentages of abdominal fat compared to the other varieties either in F1 or F2 (*P* < 0.05). Despite there being no significant variations in the ovary weights either among different quail varieties or generations, the F2 birds in all quail varieties showed higher weights of testicle and oviduct compared to those of F1 (*P* < 0.05).

**Table 7:** Carcass characteristcs of the four japanese quail varieties (BB, WW, BW, and WB)

		Live body weight	Weight after slaughtering	Carcass %	**L**iver weight (%)	Heart weight (%)	Intestinal weight (%)	Abdominal (%)	Gizzard (%)	Ovarian	Testis weight	Oviduct weight
F1	BB	270.00 ±13.88^ab^	256.88±12.9^b^	72.16±1.35^bc^	2.79± 0.17^abc^	0.92 ±0.05^c^	5.27±0.18^a^	1.05±0.1^b^	2.38 ±0.13^ab^	14.50 ±5.33^a^	8.25±1.1^abc^	9.75±2.59^b^
	WW	271.43 ±9.61^b^	252.14± 9.3^b^	75.75 ± 1.47^ab^	2.63±0.35^bc^	0.92 ±0.09^c^	4.68 ±0.31^ab^	1.02±0.29^b^	1.98 ±0.15^bc^	9.66 ±2.66^a^	6.50±0.95^bc^	10.00±1.00^b^
	BW	256.25 ±9.24^b^	246.88±9.4^b^	73.14 ± 1.38^abc^	2.65±0.2^bc^	1.17±0.06^bc^	5.19± 0.29^a^	1.48 ±0.34^b^	2.67 ±0.15^ab^	7.00±3.18^a^	7.50±0.86^abc^	7.75±3.27^b^
	WB	261.25 ±10.72^b^	249.38±10.2^b^	72.5 ± 1.58^bc^	3.57± 0.44^ab^	0.92 ±0.09^c^	5.26±0.25^a^	0.84±0.24^b^	2.22 ±0.14^bc^	11.5 ±4.48^a^	4.50± 0.5^c^	9.50±1.84^b^
Overall		264.51± 5.39	251.32	73.38	2.64	0.98	5.1	1.09	2.25	10.66	6.68	9.25
F2	BB	261.67 ±7.03^b^	245.00 ±5.2^b^	72.91 ± 1.86^bc^	2.77 ±0.43^abc^	1.49±0.2^a^	4.68 ±0.26^ab^	2.47±0.36^a^	2.7 ±0.28^ab^	15.33 ±2.72^a^	9.33±1.45^ab^	18.66±1.45^a^
	WW	265.00 ±6.83^b^	254.17 ± 6.5^b^	74.09±0.65^abc^	2.62±0.21^bc^	1.18±0.03^bc^	3.94±0.41^b^	1.57±0.19^b^	2.68 ±0.23^ab^	10.33 ±1.45^a^	11.00±1.15^a^	6.66±0.33^b^
	BW	260.83 ±8.3^b^	234.17±6.9^b^	76.77 ± 2.45^a^	3.66±0.51^a^	1.35±0.04^ab^	5.15±0.53^a^	1.53±0.29^b^	2.97 ±0.33^a^	12.00 ±1.15^a^	7.33± 1.66^abc^	66±4.05^ab^
	WB	298.33 ±10.05^a^	290±9.6^a^	71.76 ± 1.18^c^	1.91±0.47^c^	1.1±0.07^bc^	4.72 ±0.32^ab^	2.63±0.49^a^	1.68 ±0.4^c^	9.66±0.33	8.33±1.66^ab^	9.66±0.33^b^
Overall		271.45	255.83	73.88	2.74	1.28	4.62	2.05	2.03	11.83	8.99	12.16
Male		254.10^b^	240.53^b^	75.96^a^	2.35^b^	1.13^a^	4.46^b^	1.71^a^	2.31^a^	–	–	–
Female		281.48^a^	266.48^a^	71.07^b^	3.35^a^	1.09^a^	5.35^a^	1.31^a^	2.51^a^	–	–	–
*P* values												
Quail genotype		0.3158	0.4883	0.5014	0.3740	<0.0001	0.0118	<0.0001	0.2382	0.6510	0.0104	0.1050
Generation		0.1135	0.0084	0.0446	0.3771	0.0241	0.0213	0.3353	0.0032	0.4225	0.1521	0.1253
Genotype×generation interaction		0.0372	0.0058	0.1913	0.0007	0.1196	0.5436	0.0166	0.0641	0.8096	0.1728	0.0865

Results were presented as mean ± SE. Different letters in columns indicate significant differences among the groups at *P* < 0.05

### The biochemical profile

**Fig. 1:**
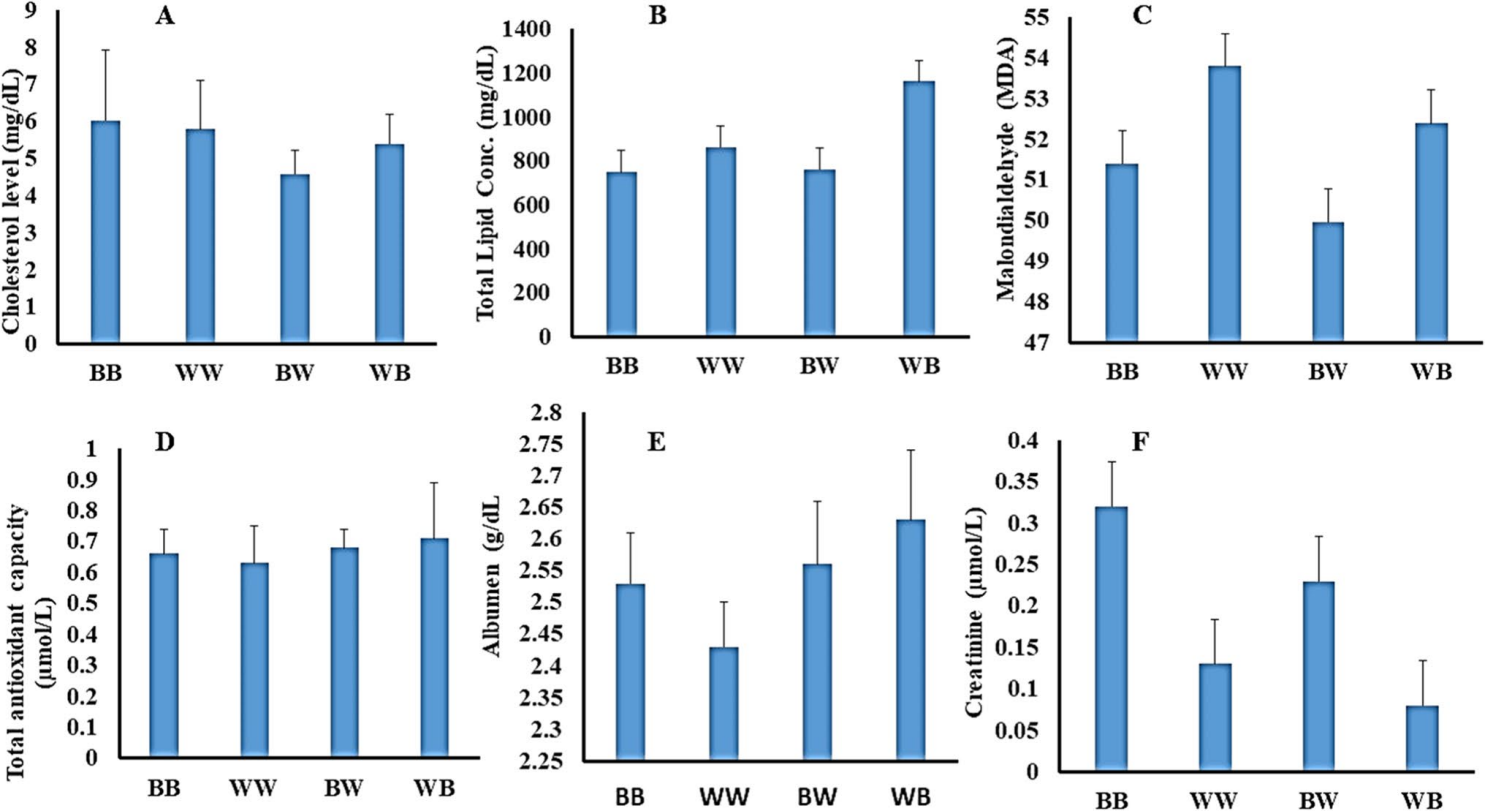
Biochemical constituents of the four japanese quail vareities (BB, WW, BW, and WB) at the end of the second generation (F2). **A** Cholestrol level (mg/dL), **B** total lipid concertation (mg/dL), **C** lipid peroxide (malondialdehyde), **D** total antioxidant capacity (μmol/L), and **E, F** albumin and creatinine concentration, respectively. Results were presented as mean ± SE

The biochemical profile of the four quail populations was evaluated at the end of the study (Fig. 1). There were no distinct observed variations among different quail populations for all assessed parameters (*P* > 0.05). However, the greatest and lowest values of cholesterol were reported for BB and BW, respectively (*P* > 0.05). The WB quails displayed the highest non-significant concentration of total lipid compared to the others (*P* > 0.05). For MDA concentration, the WW and WB quails showed the highest levels, but BB and WB showed the lowest levels (*P* > 0.05). All quail populations displayed nearly the same total antioxidant capacities (*P* > 0.05). The WB and BB exhibited higher albumen and creatinine concentrations, while WW and WB showed the lowest levels, respectively (*P* > 0.05).

### Genetic estimation

The amplification of the three studied microsatellite markers (GUJ0063, GUJ0085, and GUJ0096) was successfully done. Then, the genetic diversity of the three microsatellite loci among the four Japanese quail populations was analyzed and presented in Tables 8 and 9. However, due to the small number of markers used, these results are considered an initial clarification of genetic causes for the reported phenotypic variations. Further deep investigations depending on a larger number of markers are recommended.

**Table 8:** Number of alleles, allele size range, and heterozygosities of 3 SSRs in four quail populations

Locus	Strain	N_A_	N_e_	H_O_	H_e_	F_IS_	I	P_IC_	Chi-square	df	P value	HWE
GUJ0063	BW	2.00	2.00	0.50	0.57	0.000	0.69	0.302	0.000	1	1.00	NS
	BB	3.00	2.13	0.75	0.61	−0.412	0.90	0.521	1.440	3	0.696	NS
	WW	3.00	2.00	0.33	0.60	0.333	1.21	0.672	6.000	3	0.112	NS
	WB	4.00	2.90	0.50	0.75	0.238	1.21	0.791	5.000	6	0.554	NS
GUJ0085	BW	3.00	1.68	0.25	0.46	0.385	0.74	0.632	8.000	3	0.046	*
	BB	2.00	1.80	0.00	0.53	1.000	1.04	0.145	3.000	1	0.083	NS
	WW	2.00	1.60	0.00	0.43	1.000	0.56	0.165	4.000	1	0.046	*
	WB	3.00	2.46	0.50	0.68	0.158	0.97	0.335	1.444	3	0.695	NS
GUJ0096	BW	2.00	1.28	0.25	0.25	−0.143	0.38	0.524	0.082	1	0.775	NS
	BB^$^	1.00	1.00	0.00	0.00	Monomorphic						
	WW^$^	1.00	1.00	0.00	0.00	Monomorphic						
	WB	2.00	1.28	0.25	0.25	−0.143	0.38	0.513	0.082	1	0.775	NS
Total (mean ± SE)	BW	2.33±0.33	1.66±0.21	0.33±0.08	0.43±0.09	0.081±0.16						
	BB	2.00±0.58	1.64±0.34	0.25±0.25	0.38±0.19	0.29±0.58						
	WW	2.00±0.58	1.53±0.29	0.11±0.11	0.34±0.18	0.67±0.27						
	WB	3.00±0.58	2.22±0.49	0.42±0.08	0.56±0.16	0.084±0.12						
	Total^*^	2.33±0.26	1.76±0.17	0.28±0.07	0.43±0.07	0.242±0.135						

Total means all of 3 markers /population. Total^*^ includes all four quail populations

*N*_*A*_ the number of alleles per locus, *N*_*e*_ effective number of alleles (Kimura and Crow (1964)), *I* Shannon’s information index (Lewontin (1972)), *H*_*O*_ the observed heterozygosity, *H*_*e*_ the unbiased estimate of expected heterozygosity, *P*_*IC*_ polymorphic information content, *NS P*>0.05

*P<0.05, **P<0.01

^$^The marker GUJ0096 was monomorphic in case of the B and C quail group

**Table 9:** Nei’s measures of genetic identity and genetic distance estimates for the 3 microsatellite loci between the four studied quail populations

POP ID	BW	BB	WW	WB
BW	****	0.952	0.929	0.821
BB	0.049	****	0.854	0.873
WW	0.074	0.158	****	0.791
WB	0.198	0.136	0.234	****

Nei’s genetic identity (above diagonal) and genetic distance (below diagonal)

The total number of the observed and effective alleles (*N*_*A*_ and *Ne*, respectively) varied among quail populations. The *N*_*A*_ numbers varied between 2 and 4 for the three studied markers (GUJ0063, GUJ0085, and GUJ0096), with a total average of 2.33±0.26 alleles. The greatest *N*_*A*_ number (4) was found for the GUJ0063 marker in the case of the WB population, while the locus GUJ0096 showed a monomorphic pattern across the BB and WW quail populations. The average *N*_*A*_ of the three markers within each quail population ranged from 2.00±0.58 in the case of BB and WW to 3.00±0.58 for the WB population. These observed numbers of alleles were associated with a relatively small to moderate effective number of alleles (*N*_*e*_) ranging from 1.00 for the GUJ0096 in the case of the BB and WW quails to 2.90 for the GUJ0063 marker in the case of WB, with a total average of 1.76±0.17. Besides, the averages of *N*_*e*_ of the studied markers within each quail population were 1.66±0.21, 1.64±0.34, 1.53±0.29, and 2.22±0.49 for the BW, BB, WW, and WB, respectively. The allelic frequencies in the case of GUJ0063 and GUJ0096 ranged from 0.125 to 0.500 in the BW quails and ranged from 0.125 to 0.375 in the case of GUJ0085 in the same population. For BB quails, the allelic frequencies varied from 0.125 to 0.500, 0.167 to 0.500, and 1.00 for the GUJ0063, GUJ0085, and GUJ0096, respectively, while in the case of the WW quails, the allelic frequency ranged from 0.167 to 0.333 for the GUJ0063, 0.125 to 0.375 for the GUJ0085, and 1.00 for the GUJ0096. For the WB quails, the allelic frequencies varied from 0.125 to 0.250, 0.125 to 0.375, and 0.125 to 0.500 for the GUJ0063, GUJ0085, and GUJ0096, respectively.

The levels of the observed and expected heterozygosity were also reported. The three microsatellite loci showed variable levels of observed heterozygosity (*H*_*O*_) ranging from 0.00 for the GUJ0085 and GUJ0096 in the case of BB and WW quails to 0.75 for the GUJ0063 in the case of the BB quails, with a total average of 0.28 ±0.07. The levels of the expected heterozygosity similarly ranged from 0.00 for the GUJ0096 in the case of BB and WW quails to 0.75 for the GUJ0063 in the case of WB quails. Moreover, Shannon’s diversity index (*I*) displayed variations ranging from 0.38 in the case of BW quails for the GUJ0096 locus to 1.21 for the GUJ0063 in the case of WW and WB quails, whereas the GUJ0096 was monomorphic in the case of the BB and WW quails. The fixation coefficient (*F*_IS_) ranged from −0.412 for the GUJ0063 in the case of the BB quails to 1.00 for the GUJ0085 in the case of the BB and WW quails, with an overall average of 0.242 ± 0.135. The *F*_IS_ values of all markers within each population were 0.081±0.16, 0.29±0.58, 0.67±0.27, and 0.084±0.12 for the BW, BB, WW, and WB quails, respectively, while its values ranged from −0.143, 0.048, and 0.588 for the GUJ0096, GUJ0063, and GUJ0085, respectively.

**Fig. 2:**
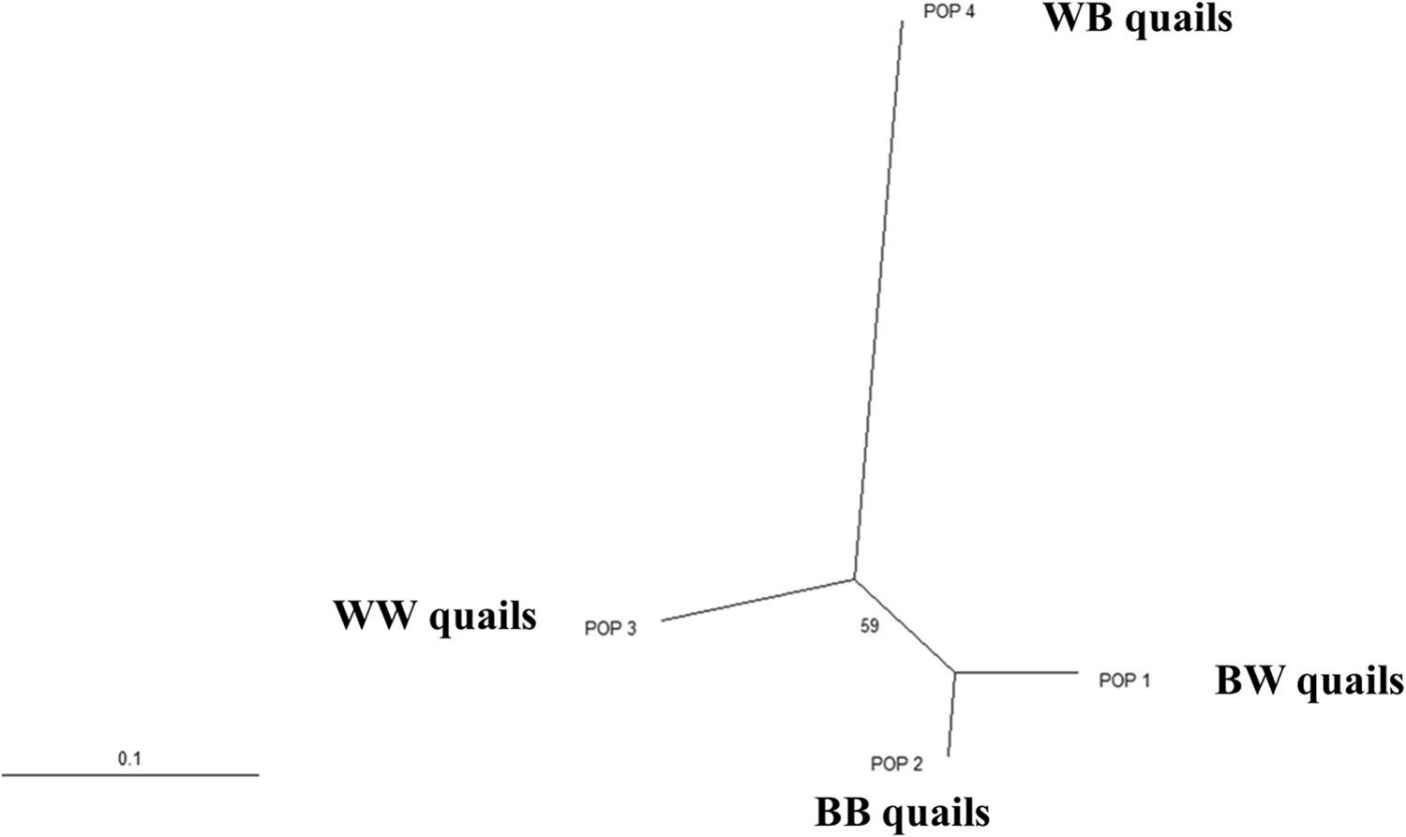
Neighbor-joining unrooted phylogenetic tree of the four Japanese quail varieties depending on 3 microsatellite markers based on Nei’s genetic distances. POP1 = BW quails, POP2 = BB quails, POP3 = WW quails, and POP4 = WB quails

Besides, the GUJ0063 (in the case of the BB, WW, and WB quails), GUJ0085 (in the case of the BW quails), and GUJ0096 (in the case of BW and WB quails) loci were highly informative markers (*P*_IC_ > 0.50) with the *P*_IC_ values ranging from 0.513 to 0.791. However, the GUJ0063 (in the case of BW quails) and GUJ0085 (in the case of the WB quails) displayed reasonably informative values (0.50 > *P*_IC_ > 0.25) with the *P*_IC_ values of 0.302 and 0.335, respectively. The GUJ0085 marker in the case of the BB and WW quails exhibited low *P*_IC_ values, 0.145 and 0.165, respectively. Moreover, from the listed values of the chi-square and *P* values of the HWE in Table 8, we concluded that the *H*_*O*_ and *H*_*e*_ did not differ significantly (*P* > 0.05) in all quail populations except the GUJ0085 in the case of the BW and WW quails which differed significantly (*P* >0.05). Table 9 presents the measures of the genetic identity and distance between the four studied quail populations. The lowest Nei genetic identity value (0.791) was recorded between the WB and WW populations, while the highest value (0.952) was found between the BW and BB quails. Regarding the genetic distance, the highest value (0.234) was between the WB and WW quails; thus, those strains were the farthest. However, the lowest value (0.049) was observed between the BW and BB quails; thus, those strains were the closest for the studied markers. These results were supported by the neighbor-joining (NJ) phylogenetic tree (Fig. 2). The tree showed a close relationship between the BW and BB quail populations. Furthermore, the BB and BW quails were clustered together and showed the most probable structure clustering, while the WW and WB populations were clustered independently confirming the high genetic distance.

## Discussion

### Phenotypic characteristics

The current study represents a physiological comparative evaluation of two different plumage color Japanese quail populations (BB and WW) and their reciprocal crossings (BW and WB) to determine the best one for meat and egg production. In this regard, we depended on several measurements such as body weights, egg number, and weight, egg quality characteristics, and carcass traits, as well as the biochemical constituents which are considered as potentially concrete bases for choosing the appropriate egg-/meat-producing population that suits the breeders’ requirements. The foremost results indicated marked variations among the BB, WW, BW, and WB quails in the aforementioned measurements during the fattening and laying period. Among these variations, the findings of body weight measurements varied among all quail populations, which agreed with Nasr et al.’s, Tavaniello et al.’s, and Maiorano et al.’s findings (Maiorano et al. 2009; Nasr et al. 2017; Tavaniello et al. 2014) who stated significant variations in body weight between different lines of Japanese quails bred for meat (broilers) and egg production (layers). These differences might be attributed to the genetic makeup of quail varieties, age, and/or nutritional management (Tavaniello 2014). Among the studied quails’ populations, WW showed the superiority of body weights which might be explained by the significant correlations between plumage color and body weight (Al-Kafajy et al. 2018; Inci et al. 2015; Nasr et al. 2017). On the other side, our findings disagreed with the findings of Tarhyel et al. (2012) who reported that white-colored quails displayed the lowest body weights. Additionally, the BW and WB came in second and third places after the WW quails, respectively. Such superiority of cross lines perhaps indicates the direct additive genetic effect of sire line on quail body weight (Aboul-Seoud et al. 2021). Besides, the heavy weights were observed in F2 compared to F1 which could be explained by the higher level of heterosis in the F2 generation that again might be associated with the genetic structure of the F2 birds (Tavaniello 2014).

When comparing the carcass characteristics among different quail populations, significant variations were observed with the highest values reported in the case of WW and BW for carcass % and most of the internal organ weights; in addition, the values of F2 were higher than that of F1. These current results are in agreement with Sharaf et al.’s (Sharaf et al. 2006) findings which reported a significant superiority of crossbred quail populations compared to the purebreds as a result of the possible positive heterosis effect, while these findings disagreed with Inci et al.’s (Inci et al. 2015) findings which stated that the white-colored quails, compared to the wild-type quail, had the lowest values of carcass traits suggesting a depressive effect of the recessive white plumage color mutation on carcass traits. Regarding the abdominal fat %, the marked highest values were noticed in the case of the BB and WB quails. This perhaps can be explained by the association of high-fat deposition with brown plumage–colored quails. The differences in the measured carcass traits may be linked with the body weight variations among different quail populations. This is probably because of the positive genetic correlations between body weight and carcass traits (Hussain et al. 2013; Ibrahim et al. 2021). Therefore, body weight is considered the concrete index in evaluating the differences among different animal and poultry species (Ahmad et al. 2018; Hussain et al. 2013; Khaldari et al. 2010).

For the egg number, there were no clear superiorities of any of the studied quail populations through the recorded period of production. However, there were clear advantages of BB at the beginning of egg production, and then, the cross lines showed higher performance. These findings might come in agreement with Bed’hom et al.’s and Inci et al.’s (Bed’hom et al. 2012; Inci et al. 2015) findings who did not report any superiority of any different colored-quail populations. For egg weight, the values of the first hatch (F1) were higher, for all quail populations, than the second one (F2). Our findings were comparable with those of Ashok and Reddy and Lan et al. (Ashok and Reddy 2010; Lan et al. 2021) who stated an association between egg weight and different quail genotypes. This might be explained by the superior nongenetic effects on weights. Upon examining the effect of quail varieties and generation on egg quality characteristics including albumen height, albumen width, albumen weight, yolk width, yolk height, yolk color, yolk weight, the Haugh unit (HU), and eggshell thickness, along with shape, albumin, and yolk indices, significant variations were found. In general, the BW and WB had the highest values, relative to the others, and most of the assessed parameters and values of F2 were higher than that of F1. These findings might be explained with the significant association of feather color with egg quality characteristics in Japanese quail population (Al-Kafajy et al. 2018; Inci et al. 2015; Lan et al. 2021). Besides, for biochemical parameter evaluations, there were no clear variations among the studied quail populations. These findings have come in line with Al-Kafajy et al.’s results who reported non-significant differences among different plumage-colored quails in terms of cholesterol, total lipids, and triglycerides (Al-Kafajy et al. 2018).

The summary of the phenotypic evaluation indicated the presence of contradictory findings on the studied traits, with the previous studies, among the different plumage color quails, whereas some authors have proved no differences, while others established significant differences. This is owing to the complications to compare body weights, carcass, and egg quality results with those reported in other studies, because of many reasons such as the genetic structure, environmental and managerial conditions, considered statistical factors, feeding regime, and age of slaughtering (Vali 2008).

### Genetic evaluation

Despite the obtained results, the three studied microsatellite markers could assist in genetically explaining the detected physiological variations in the four quail populations: BB, WW, BW, and WB in terms of body weights, carcass characteristics, and egg production and quality features. However, this explanation may be inadequate, and more markers are recommended for the future explanation since results are varied depending on the number of the studied microsatellite markers.

The genetic variation can be assessed depending on many genetic diversity indices such as the observed and the effective number of alleles (*N*_*A*_ and *N*_*e*_, respectively) as they give an indication on the difference in allele frequency in a population (Abdel-Kafy et al. 2021). In this regard, the average number of alleles ranged from 1 to 4 with a range of *N*_*e*_ form 1.00 to 2.90. This small range of *N*_*A*_ probably explains the less genetic variation among the studied quail populations which might be explained by the small sample size of those quail populations that might predispose the genetic drift and inbreeding (Abdel-Kafy et al. 2021). Although there were small *N*_*A*_ and *N*_*e*_ ranges which might indicate low genetic variation, the level of *H*_*O*_ and *H*_*e*_ varied from 0.00 to 0.75 without deviation from HWE in most of the studied quail populations indicating that these quail populations were in HWE. However, the WW and BW quails had the lowest level of heterozygosity as confirmed by low values of *H*_*O*_ (0.00 and 0.25, respectively) and a significant deviation from the Hardy-Weinberg equilibrium (HWE) in case GUJ0085, which might indicate a possibility of a non-random mating and inbreeding as confirmed by less heterozygosity (Abdel-Kafy et al. 2021) and the high positive values of heterozygosity deficit index (*F*_IS_). This high levels of inbreeding in these population might be risky as it could cause genetic diseases and negatively influence bird’s performance (Sharma et al. 2016). Our findings agreed with Ibrahim et al. and Kayang et al. (Ibrahim et al. 2021; Kayang et al. 2002) who reported nearly similar number of alleles, *H*_*O*_ and *H*_*e*_ values. However, our findings disagreed with the previous studies which were conducted on different quail genotypes by Bai et al. (Bai et al. 2013; Bai et al. 2018), Kawahara-Miki et al. (Kawahara-Miki et al. 2013), and Shimma and Tadano (Shimma and Tadano 2019) which reported higher number of alleles. In summary, the incomparable results in most of the diversity estimates from different studies are probably because of using different microsatellite sets as well as the number of the analysed markers (Sharma et al. 2016). Additionally, the differences in allele numbers (*N*_*A*_ and *N*_*e*_) between the current and previous investigations may be linked with the differences in sample size, population structure, number of markers used, population-specific alleles and/or allele scoring bias (null allele or allele drop out), and sampling strategy (Rashid et al. 2020). Thus, more microsatellite markers are recommended for further explanation among these quail populations.

Besides, the values of the polymorphic information content (*P*_IC_) suggested that most of the used microsatellite loci were variably polymorphic and informative ( 0.20 > *P*_IC_ > 0.50) according to the classification by Botstein et al. (Botstein et al. 1980). However, GUJ0096 marker showed monomorphic pattern in BB and WW quails. The high *P*_IC_ values (> 0.50) for GUJ0063 in case of BB, WW, and WB, those for GUJ0085 in case of BW, and for GUJ0096 in case of BW and WB might suggest a high degree of polymorphism between the studied quail populations and the possibility of a progeny to acquire some allelic markers from its parents (Ibrahim et al. 2021). This finding requires further deep confirmation using more microsatellite and other genetic markers. In addition, the stated high *P*_IC_ values of the studied markers possibly explain the potential role of these markers for population assignment as well as genome mapping studies in addition to genetic diversity analysis (Sharma et al. 2016). Nevertheless, this is not enough for validating the genetic diversity among the studied quail populations, and further investigations using more markers are recommended. Thus, collectively, from the results of *H*_*O*_, *P*_IC_, and Shannon’s information index, we can conclude that the selected 3 microsatellites markers, used in this study, might present a possible assessment of the genetic diversity in the different quail populations (Rashid et al. 2020).

Moreover, the BB and BW quail populations might be closely related based on the reported low values of genetic distance and high genetic identity. This probable low genetic differentiation between these two quail populations might designate a recent gene flow among themselves as well as common ancestors for constructing the populations (Rashid et al. 2020). However, again, this finding demands more detailed explanation using more microsatellite markers.

## Conclusion

The current investigation presented the physiological variations and their possible preliminary genetic explanation between BB and WW and their reciprocal crosses, WB and BW, in live body weight, carcass characteristics, egg weights, and quality. In conclusion, the WW quails exhibited the heaviest weights followed by BW, WB, and BB, respectively. Besides, the F2 populations showed higher weights compared to the F1. Additionally, the WW and BW quails possessed higher percentages for the carcass, and most of the internal organ weights and the values of F2 were higher than that of F1. However, the BB and WB had the highest values of abdominal fat %. The obtained results might introduce an initial scientific basis for evaluating and employing the genetic properties of BB, WW, BW, and WB quails in the genetic improvement of the Japanese quail to overcome inbreeding in quail farming and, accordingly, improve its performance. Further deep analysis of the genetic diversity among these quail populations using more microsatellite markers is recommended.

## Data Availability

The authors acknowledge that the data presented in this study must be deposited and made publicly available in an acceptable repository, prior to publication.
